# [4-(Di-*tert*-butyl­fluoro­silan­yl)phenyl]methanol

**DOI:** 10.1107/S160053681003148X

**Published:** 2010-08-11

**Authors:** Ljuba Iovkova-Berends, Christina Dietz, Klaus Jurkschat

**Affiliations:** a Fakultät Chemie, Technische Universität Dortmund, 44221 Dortmund, Germany

## Abstract

The asymmetric unit of the title compound, C_15_H_25_FOSi, contains two independent mol­ecules. Each of the Si atoms approximates the expected tetra­hedral geometry with Si—F bond lengths of 1.6128 (11) and 1.6068 (11) Å in the two independent mol­ecules. In the crystal, supra­molecular chains along *a* are found mediated by O—H⋯O hydrogen bonds.

## Related literature

For synthetic background, see: Iovkova *et al.* (2009 ▶). For related structures, see: Iovkova *et al.* (2009 ▶); Bradtmöller *et al.* (2006 ▶).
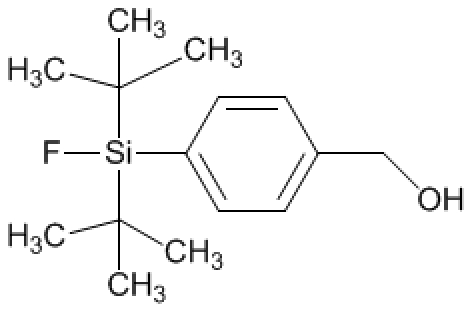


## Experimental

### 

#### Crystal data

C_15_H_25_FOSi
*M* *_r_* = 268.44
Triclinic, 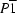

*a* = 8.3213 (5) Å
*b* = 12.4011 (7) Å
*c* = 17.1036 (9) Å
α = 103.581 (5)°
β = 101.553 (5)°
γ = 107.487 (5)°
*V* = 1564.96 (18) Å^3^
*Z* = 4
Mo *K*α radiation
μ = 0.15 mm^−1^
*T* = 173 K
0.26 × 0.14 × 0.12 mm

#### Data collection

Xcalibur2 CCD diffractometer
Absorption correction: multi-scan (*CrysAlis RED*; Oxford Diffraction, 2008 ▶) *T*_min_ = 0.928, *T*_max_ = 1.000
5698 measured reflections
5698 independent reflections
3074 reflections with *I* > 2σ(*I*)
*R*_int_ = 0.042

#### Refinement

*R*[*F* ^2^ > 2σ(*F* ^2^)] = 0.037
*wR*(*F* ^2^) = 0.070
*S* = 0.81
5698 reflections
337 parameters
H atoms treated by a mixture of independent and constrained refinement
Δρ_max_ = 0.27 e Å^−3^
Δρ_min_ = −0.24 e Å^−3^

### 

Data collection: *CrysAlis CCD* (Oxford Diffraction, 2008 ▶); cell refinement: *CrysAlis RED* (Oxford Diffraction, 2008 ▶); data reduction: *CrysAlis RED*; program(s) used to solve structure: *SHELXS97* (Sheldrick, 2008 ▶); program(s) used to refine structure: *SHELXL97* (Sheldrick, 2008 ▶); molecular graphics: *DIAMOND* (Brandenburg, 2006 ▶); software used to prepare material for publication: *publCIF* (Westrip, 2010 ▶).

## Supplementary Material

Crystal structure: contains datablocks global, I. DOI: 10.1107/S160053681003148X/tk2698sup1.cif

Structure factors: contains datablocks I. DOI: 10.1107/S160053681003148X/tk2698Isup2.hkl

Additional supplementary materials:  crystallographic information; 3D view; checkCIF report

## Figures and Tables

**Table 1:** Hydrogen-bond geometry (Å, °)

*D*—H⋯*A*	*D*—H	H⋯*A*	*D*⋯*A*	*D*—H⋯*A*
O7—H7*A*⋯O27^i^	0.74 (5)	2.01 (5)	2.707 (3)	157 (5)
O27—H27*A*⋯O27^ii^	0.76 (3)	2.00 (3)	2.727 (3)	160 (5)
O7—H7*B*⋯O7^iii^	0.70 (4)	2.14 (3)	2.787 (4)	154 (5)
O27—H27*B*⋯O7^iv^	0.80 (4)	1.92 (5)	2.707 (3)	167 (4)

Symmetry codes: (i) 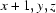; (ii) 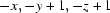; (iii) 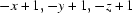; (iv) 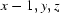.

**Fig. 1.**
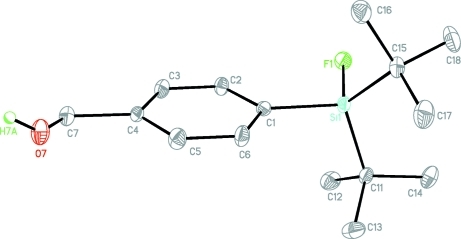
The molecular structure of the first independent molecule in (I) showing the atom-labelling scheme and displacement ellipsoids at the 30% probability level. Only one orientation of the disordered H atom of the OH group (bound to C7) is shown and the remaining H atoms have been deleted.

**Fig. 2.**
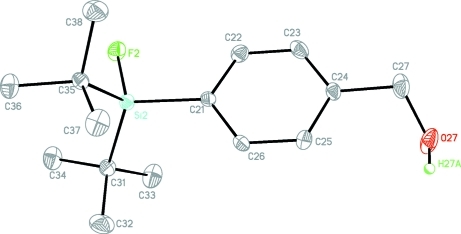
The molecular structure of the second independent molecule in (I) showing the atom-labelling scheme and displacement ellipsoids at the 30% probability level. Only one orientation of the disordered H atom of the OH group (bound to C27) is shown and the remaining H atoms have been deleted.

**Fig. 3.**
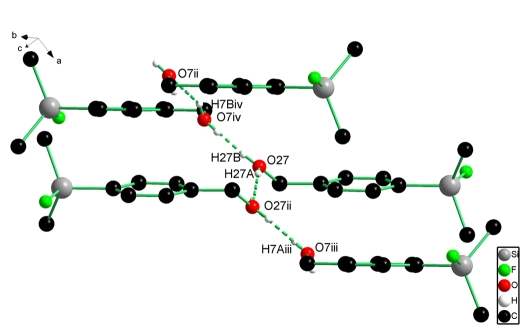
Supramolecular chain in the crystal of (I). One dimensional polymer is formed in a direction via OH···O-bridges. Each of the displayed H atoms is present 50% of the time and other H atoms have been omitted.

## supplementary crystallographic information

### Comment

In the title compound, (I), the Si atoms show a distorted tetrahedral configuration, with bond angles ranging from 104.23 (7) ° (F2—Si2—C21) to 118.45 (9) ° (C31—Si2—C35). The Si—F bond lengths of 1.6128 (11) Å (Si1—F1) and 1.6068 (11) Å (Si2—F2) are comparable with that in *tert*-butylfluorodiphenylsilane, Si—F = 1.6004 (10) Å (Bradtmöller *et al.*, 2006). In the crystal packing, molecules aggregate into supramolecular chains along *a* via O–H···O hydrogen bonding, Table 1 and Fig. 2.

### Experimental

The title compound was prepared according to a literature procedure (Iovkova *et al.*, 2009). The crude product was recrystallized from hexane to give colourless crystals.

### Refinement

The C-bound H atoms were geometrically placed (C–H = 0.93–0.96 Å) and refined as riding with *U*_iso_(H) = 1.2Ueq(C). The O—H atoms were located in a difference Fourier map but for each OH-groups, this was disordered over two positions of equal weight (from isotropic refinement).

### Crystal data

**Table:**

C_15_H_25_FOSi	*F*(000) = 584
*M**_r_* = 268.44	*D*_x_ = 1.139 Mg m^−^^3^
Triclinic, *P*1	Melting point: 355 K
*a* = 8.3213 (5) Å	Mo *K*α radiation, λ = 0.71073 Å
*b* = 12.4011 (7) Å	Cell parameters from 5698 reflections
*c* = 17.1036 (9) Å	θ = 2.5–25.5°
α = 103.581 (5)°	µ = 0.15 mm^−^^1^
β = 101.553 (5)°	*T* = 173 K
γ = 107.487 (5)°	Block, colourless
*V* = 1564.96 (18) Å^3^	0.26 × 0.14 × 0.12 mm
*Z* = 4	

### Data collection

**Table:**

Xcalibur2 CCD diffractometer	5698 independent reflections
Radiation source: Enhance (Mo) X-ray Source	3074 reflections with *I* > 2σ(*I*)
graphite	*R*_int_ = 0.042
Detector resolution: 16.0560 pixels mm^-1^	θ_max_ = 25.5°, θ_min_ = 2.5°
489 frames via ω–rotation (Δω = 1°) and two times 40 s per frame (8 sets at different κ–angles) scans	*h* = −10→9
Absorption correction: multi-scan (*CrysAlis RED*; Oxford Diffraction, 2008)	*k* = −15→14
*T*_min_ = 0.928, *T*_max_ = 1.000	*l* = 0→20
5698 measured reflections	

### Refinement

**Table:**

Refinement on *F*^2^	Primary atom site location: structure-invariant direct methods
Least-squares matrix: full	Secondary atom site location: difference Fourier map
*R*[*F*^2^ > 2σ(*F*^2^)] = 0.037	Hydrogen site location: inferred from neighbouring sites
*wR*(*F*^2^) = 0.070	H atoms treated by a mixture of independent and constrained refinement
*S* = 0.81	*w* = 1/[σ^2^(*F*_o_^2^) + (0.0317*P*)^2^] where *P* = (*F*_o_^2^ + 2*F*_c_^2^)/3
5698 reflections	(Δ/σ)_max_ = 0.001
337 parameters	Δρ_max_ = 0.27 e Å^−^^3^
0 restraints	Δρ_min_ = −0.24 e Å^−^^3^

### Special details

**Table:**

Geometry. All e.s.d.'s (except the e.s.d. in the dihedral angle between two l.s. planes) are estimated using the full covariance matrix. The cell e.s.d.'s are taken into account individually in the estimation of e.s.d.'s in distances, angles and torsion angles; correlations between e.s.d.'s in cell parameters are only used when they are defined by crystal symmetry. An approximate (isotropic) treatment of cell e.s.d.'s is used for estimating e.s.d.'s involving l.s. planes.
Refinement. Refinement of *F*^2^ against ALL reflections. The weighted *R*-factor *wR* and goodness of fit *S* are based on *F*^2^, conventional *R*-factors *R* are based on *F*, with *F* set to zero for negative *F*^2^. The threshold expression of *F*^2^ > σ(*F*^2^) is used only for calculating *R*-factors(gt) *etc*. and is not relevant to the choice of reflections for refinement. *R*-factors based on *F*^2^ are statistically about twice as large as those based on *F*, and *R*- factors based on ALL data will be even larger.

### Fractional atomic coordinates and isotropic or equivalent isotropic displacement parameters (Å^2^)

**Table:**

	*x*	*y*	*z*	*U*_iso_*/*U*_eq_	Occ. (<1)
Si1	0.21087 (9)	0.53468 (5)	0.86407 (4)	0.02174 (17)	
Si2	0.14914 (9)	−0.00184 (5)	0.27572 (4)	0.02178 (17)	
F1	0.20462 (15)	0.44023 (10)	0.91521 (7)	0.0292 (3)	
F2	0.05148 (16)	−0.13240 (9)	0.28033 (7)	0.0329 (3)	
O7	0.5783 (3)	0.43277 (19)	0.53749 (11)	0.0423 (6)	
O27	−0.1288 (3)	0.40525 (16)	0.50475 (12)	0.0451 (6)	
H7A	0.660 (6)	0.441 (4)	0.525 (3)	0.026*	0.50
H27A	−0.074 (5)	0.456 (3)	0.492 (3)	0.025*	0.50
H7B	0.519 (6)	0.447 (4)	0.510 (3)	0.017*	0.50
H27B	−0.213 (6)	0.408 (3)	0.520 (2)	0.015*	0.50
C1	0.2944 (3)	0.48053 (18)	0.77482 (12)	0.0195 (5)	
C2	0.3008 (3)	0.36675 (18)	0.75402 (12)	0.0233 (5)	
H2A	0.2634	0.3177	0.7855	0.028*	
C3	0.3610 (3)	0.32447 (18)	0.68797 (13)	0.0259 (6)	
H3A	0.3619	0.2475	0.6755	0.031*	
C4	0.4204 (3)	0.39537 (19)	0.64003 (12)	0.0215 (5)	
C5	0.4156 (3)	0.50816 (19)	0.65974 (13)	0.0296 (6)	
H5A	0.4551	0.5573	0.6286	0.035*	
C6	0.3529 (3)	0.54993 (18)	0.72536 (13)	0.0280 (6)	
H6A	0.3498	0.6263	0.7367	0.034*	
C7	0.4856 (3)	0.3454 (2)	0.56882 (13)	0.0325 (6)	
H7D	0.5621	0.3055	0.5882	0.039*	
H7C	0.3856	0.2862	0.5234	0.039*	
C11	−0.0251 (3)	0.51978 (18)	0.82264 (13)	0.0239 (5)	
C12	−0.1355 (3)	0.38556 (19)	0.78331 (14)	0.0375 (6)	
H12A	−0.2566	0.3745	0.7601	0.056*	
H12B	−0.1266	0.3472	0.8258	0.056*	
H12C	−0.0923	0.3513	0.7395	0.056*	
C13	−0.0466 (3)	0.5789 (2)	0.75359 (14)	0.0400 (7)	
H13A	−0.1687	0.5664	0.7317	0.060*	
H13B	−0.0053	0.5444	0.7091	0.060*	
H13C	0.0207	0.6629	0.7769	0.060*	
C14	−0.0985 (3)	0.5731 (2)	0.89152 (14)	0.0403 (7)	
H14A	−0.2219	0.5556	0.8681	0.060*	
H14B	−0.0373	0.6579	0.9133	0.060*	
H14C	−0.0823	0.5391	0.9361	0.060*	
C15	0.3711 (3)	0.68180 (18)	0.94140 (13)	0.0271 (6)	
C16	0.5595 (3)	0.6837 (2)	0.94900 (14)	0.0395 (6)	
H16A	0.6419	0.7539	0.9930	0.059*	
H16B	0.5850	0.6840	0.8967	0.059*	
H16C	0.5691	0.6142	0.9620	0.059*	
C17	0.3552 (3)	0.78949 (19)	0.91528 (14)	0.0437 (7)	
H17A	0.4439	0.8614	0.9551	0.066*	
H17B	0.2407	0.7923	0.9140	0.066*	
H17C	0.3710	0.7820	0.8604	0.066*	
C18	0.3433 (3)	0.6950 (2)	1.02885 (13)	0.0445 (7)	
H18A	0.4252	0.7703	1.0676	0.067*	
H18B	0.3620	0.6317	1.0479	0.067*	
H18C	0.2251	0.6914	1.0256	0.067*	
C21	0.0847 (3)	0.09866 (18)	0.35247 (12)	0.0212 (5)	
C22	−0.0082 (3)	0.05502 (19)	0.40438 (13)	0.0285 (6)	
H22	−0.0377	−0.0251	0.4002	0.034*	
C23	−0.0583 (3)	0.1263 (2)	0.46184 (13)	0.0319 (6)	
H23	−0.1194	0.0935	0.4957	0.038*	
C24	−0.0194 (3)	0.24503 (19)	0.47002 (13)	0.0251 (6)	
C25	0.0705 (3)	0.29039 (19)	0.41831 (13)	0.0320 (6)	
H25	0.0965	0.3700	0.4218	0.038*	
C26	0.1225 (3)	0.21910 (18)	0.36147 (12)	0.0276 (6)	
H26	0.1846	0.2525	0.3281	0.033*	
C27	−0.0724 (3)	0.3224 (2)	0.53407 (14)	0.0399 (7)	
H27C	−0.1666	0.2721	0.5495	0.048*	
H27D	0.0272	0.3648	0.5843	0.048*	
C31	0.0532 (3)	−0.00873 (18)	0.16479 (12)	0.0264 (5)	
C32	0.1582 (3)	0.0998 (2)	0.14295 (13)	0.0487 (7)	
H32A	0.2772	0.1034	0.1497	0.073*	
H32B	0.1581	0.1713	0.1798	0.073*	
H32C	0.1046	0.0922	0.0858	0.073*	
C33	−0.1372 (3)	−0.0130 (2)	0.15359 (14)	0.0466 (7)	
H33A	−0.2040	−0.0803	0.1671	0.070*	
H33B	−0.1898	−0.0203	0.0963	0.070*	
H33C	−0.1363	0.0589	0.1904	0.070*	
C34	0.0475 (3)	−0.1222 (2)	0.10223 (13)	0.0426 (7)	
H34A	−0.0180	−0.1908	0.1149	0.064*	
H34B	0.1654	−0.1200	0.1067	0.064*	
H34C	−0.0084	−0.1268	0.0461	0.064*	
C35	0.3920 (3)	0.02815 (18)	0.31370 (12)	0.0226 (5)	
C36	0.4563 (3)	−0.0465 (2)	0.25128 (14)	0.0396 (7)	
H36A	0.3878	−0.1295	0.2381	0.059*	
H36B	0.5781	−0.0321	0.2758	0.059*	
H36C	0.4432	−0.0247	0.2007	0.059*	
C37	0.5025 (3)	0.15982 (19)	0.33339 (14)	0.0395 (6)	
H37A	0.4636	0.2078	0.3725	0.059*	
H37B	0.4888	0.1806	0.2825	0.059*	
H37C	0.6243	0.1736	0.3575	0.059*	
C38	0.4238 (3)	−0.0041 (2)	0.39535 (14)	0.0447 (7)	
H38A	0.3555	−0.0865	0.3842	0.067*	
H38B	0.3894	0.0445	0.4363	0.067*	
H38C	0.5466	0.0098	0.4166	0.067*	

### Atomic displacement parameters (Å^2^)

**Table:**

	*U*^11^	*U*^22^	*U*^33^	*U*^12^	*U*^13^	*U*^23^
Si1	0.0253 (4)	0.0237 (4)	0.0206 (4)	0.0121 (3)	0.0116 (3)	0.0066 (3)
Si2	0.0236 (4)	0.0189 (4)	0.0209 (4)	0.0072 (3)	0.0062 (3)	0.0039 (3)
F1	0.0361 (9)	0.0327 (7)	0.0269 (7)	0.0165 (7)	0.0139 (7)	0.0150 (6)
F2	0.0346 (9)	0.0207 (7)	0.0438 (8)	0.0079 (7)	0.0158 (7)	0.0103 (6)
O7	0.0370 (15)	0.0663 (13)	0.0395 (13)	0.0232 (11)	0.0288 (11)	0.0246 (10)
O27	0.0452 (14)	0.0305 (12)	0.0754 (15)	0.0197 (11)	0.0415 (12)	0.0170 (10)
C1	0.0163 (13)	0.0234 (13)	0.0196 (12)	0.0072 (11)	0.0062 (11)	0.0073 (10)
C2	0.0263 (15)	0.0230 (13)	0.0254 (13)	0.0091 (12)	0.0149 (12)	0.0101 (11)
C3	0.0264 (15)	0.0213 (13)	0.0298 (14)	0.0102 (12)	0.0106 (12)	0.0035 (11)
C4	0.0155 (14)	0.0312 (14)	0.0190 (12)	0.0107 (11)	0.0070 (11)	0.0053 (11)
C5	0.0349 (16)	0.0336 (15)	0.0322 (14)	0.0161 (13)	0.0205 (13)	0.0182 (12)
C6	0.0383 (17)	0.0234 (13)	0.0294 (14)	0.0173 (13)	0.0163 (13)	0.0076 (11)
C7	0.0378 (17)	0.0440 (16)	0.0269 (14)	0.0253 (14)	0.0162 (13)	0.0121 (12)
C11	0.0287 (15)	0.0222 (13)	0.0260 (13)	0.0137 (12)	0.0116 (12)	0.0084 (11)
C12	0.0235 (16)	0.0381 (16)	0.0454 (16)	0.0085 (13)	0.0065 (13)	0.0101 (13)
C13	0.0327 (17)	0.0486 (17)	0.0431 (16)	0.0206 (14)	0.0060 (14)	0.0189 (14)
C14	0.0360 (18)	0.0456 (16)	0.0499 (17)	0.0237 (14)	0.0239 (15)	0.0130 (13)
C15	0.0295 (15)	0.0238 (13)	0.0273 (14)	0.0099 (12)	0.0124 (12)	0.0031 (11)
C16	0.0307 (17)	0.0383 (15)	0.0377 (15)	0.0089 (13)	0.0068 (13)	−0.0017 (12)
C17	0.0462 (19)	0.0288 (15)	0.0499 (17)	0.0106 (14)	0.0173 (15)	0.0025 (13)
C18	0.0477 (19)	0.0450 (17)	0.0286 (15)	0.0115 (15)	0.0133 (14)	−0.0050 (12)
C21	0.0217 (14)	0.0258 (14)	0.0173 (12)	0.0109 (11)	0.0037 (11)	0.0077 (10)
C22	0.0283 (15)	0.0249 (13)	0.0330 (14)	0.0096 (12)	0.0092 (13)	0.0103 (12)
C23	0.0263 (16)	0.0484 (17)	0.0275 (14)	0.0163 (13)	0.0134 (13)	0.0158 (13)
C24	0.0247 (15)	0.0349 (15)	0.0202 (13)	0.0178 (12)	0.0072 (12)	0.0071 (11)
C25	0.0435 (17)	0.0268 (14)	0.0307 (14)	0.0189 (13)	0.0142 (13)	0.0070 (12)
C26	0.0383 (17)	0.0277 (15)	0.0234 (13)	0.0133 (13)	0.0169 (13)	0.0116 (11)
C27	0.0365 (18)	0.0518 (17)	0.0350 (16)	0.0263 (15)	0.0126 (14)	0.0051 (13)
C31	0.0300 (15)	0.0261 (14)	0.0183 (12)	0.0101 (12)	0.0023 (11)	0.0028 (10)
C32	0.067 (2)	0.0525 (18)	0.0264 (15)	0.0215 (16)	0.0095 (15)	0.0157 (13)
C33	0.0416 (18)	0.0554 (18)	0.0326 (15)	0.0267 (15)	−0.0075 (14)	−0.0004 (13)
C34	0.0434 (18)	0.0502 (17)	0.0240 (14)	0.0211 (15)	−0.0011 (13)	−0.0029 (12)
C35	0.0231 (15)	0.0227 (13)	0.0200 (13)	0.0063 (12)	0.0067 (12)	0.0055 (10)
C36	0.0311 (17)	0.0461 (16)	0.0456 (16)	0.0189 (14)	0.0148 (14)	0.0123 (13)
C37	0.0265 (16)	0.0366 (16)	0.0409 (16)	0.0061 (13)	−0.0006 (13)	0.0022 (12)
C38	0.0347 (18)	0.0645 (19)	0.0401 (16)	0.0214 (15)	0.0070 (14)	0.0247 (14)

### Geometric parameters (Å, °)

**Table:**

Si1—F1	1.6131 (11)	C16—H16C	0.9600
Si1—C1	1.8677 (19)	C17—H17A	0.9600
Si1—C15	1.885 (2)	C17—H17B	0.9600
Si1—C11	1.886 (2)	C17—H17C	0.9600
Si2—F2	1.6072 (11)	C18—H18A	0.9600
Si2—C21	1.865 (2)	C18—H18B	0.9600
Si2—C31	1.876 (2)	C18—H18C	0.9600
Si2—C35	1.885 (2)	C21—C22	1.389 (2)
O7—C7	1.412 (3)	C21—C26	1.394 (3)
O7—H7A	0.74 (5)	C22—C23	1.377 (3)
O7—H7B	0.70 (4)	C22—H22	0.9300
O27—C27	1.402 (3)	C23—C24	1.376 (3)
O27—H27A	0.76 (3)	C23—H23	0.9300
O27—H27B	0.80 (4)	C24—C25	1.380 (3)
C1—C2	1.392 (3)	C24—C27	1.506 (3)
C1—C6	1.396 (2)	C25—C26	1.381 (3)
C2—C3	1.382 (2)	C25—H25	0.9300
C2—H2A	0.9300	C26—H26	0.9300
C3—C4	1.389 (2)	C27—H27C	0.9700
C3—H3A	0.9300	C27—H27D	0.9700
C4—C5	1.374 (3)	C31—C34	1.536 (3)
C4—C7	1.508 (3)	C31—C32	1.540 (3)
C5—C6	1.387 (3)	C31—C33	1.540 (3)
C5—H5A	0.9300	C32—H32A	0.9600
C6—H6A	0.9300	C32—H32B	0.9600
C7—H7D	0.9700	C32—H32C	0.9600
C7—H7C	0.9700	C33—H33A	0.9600
C11—C14	1.538 (3)	C33—H33B	0.9600
C11—C13	1.538 (3)	C33—H33C	0.9600
C11—C12	1.541 (3)	C34—H34A	0.9600
C12—H12A	0.9600	C34—H34B	0.9600
C12—H12B	0.9600	C34—H34C	0.9600
C12—H12C	0.9600	C35—C37	1.530 (3)
C13—H13A	0.9600	C35—C36	1.531 (3)
C13—H13B	0.9600	C35—C38	1.535 (2)
C13—H13C	0.9600	C36—H36A	0.9600
C14—H14A	0.9600	C36—H36B	0.9600
C14—H14B	0.9600	C36—H36C	0.9600
C14—H14C	0.9600	C37—H37A	0.9600
C15—C17	1.537 (3)	C37—H37B	0.9600
C15—C18	1.538 (3)	C37—H37C	0.9600
C15—C16	1.540 (3)	C38—H38A	0.9600
C16—H16A	0.9600	C38—H38B	0.9600
C16—H16B	0.9600	C38—H38C	0.9600
			
F1—Si1—C1	104.81 (8)	C15—C17—H17C	109.5
F1—Si1—C15	104.91 (8)	H17A—C17—H17C	109.5
C1—Si1—C15	112.43 (9)	H17B—C17—H17C	109.5
F1—Si1—C11	105.55 (8)	C15—C18—H18A	109.5
C1—Si1—C11	109.86 (9)	C15—C18—H18B	109.5
C15—Si1—C11	118.05 (10)	H18A—C18—H18B	109.5
F2—Si2—C21	104.23 (8)	C15—C18—H18C	109.5
F2—Si2—C31	105.14 (8)	H18A—C18—H18C	109.5
C21—Si2—C31	111.74 (9)	H18B—C18—H18C	109.5
F2—Si2—C35	105.42 (8)	C22—C21—C26	115.72 (18)
C21—Si2—C35	110.49 (10)	C22—C21—Si2	120.48 (15)
C31—Si2—C35	118.46 (9)	C26—C21—Si2	123.79 (14)
C7—O7—H7A	131 (3)	C23—C22—C21	122.27 (19)
C7—O7—H7B	111 (4)	C23—C22—H22	118.9
H7A—O7—H7B	109 (5)	C21—C22—H22	118.9
C27—O27—H27A	127 (4)	C24—C23—C22	121.22 (18)
C27—O27—H27B	108 (3)	C24—C23—H23	119.4
H27A—O27—H27B	122 (5)	C22—C23—H23	119.4
C2—C1—C6	116.25 (17)	C23—C24—C25	117.76 (19)
C2—C1—Si1	120.95 (14)	C23—C24—C27	120.68 (18)
C6—C1—Si1	122.80 (15)	C25—C24—C27	121.56 (19)
C3—C2—C1	122.00 (17)	C24—C25—C26	120.90 (19)
C3—C2—H2A	119.0	C24—C25—H25	119.5
C1—C2—H2A	119.0	C26—C25—H25	119.5
C2—C3—C4	120.86 (19)	C25—C26—C21	122.12 (18)
C2—C3—H3A	119.6	C25—C26—H26	118.9
C4—C3—H3A	119.6	C21—C26—H26	118.9
C5—C4—C3	118.02 (17)	O27—C27—C24	112.91 (17)
C5—C4—C7	123.09 (17)	O27—C27—H27C	109.0
C3—C4—C7	118.89 (18)	C24—C27—H27C	109.0
C4—C5—C6	121.05 (18)	O27—C27—H27D	109.0
C4—C5—H5A	119.5	C24—C27—H27D	109.0
C6—C5—H5A	119.5	H27C—C27—H27D	107.8
C5—C6—C1	121.81 (19)	C34—C31—C32	108.43 (17)
C5—C6—H6A	119.1	C34—C31—C33	108.07 (19)
C1—C6—H6A	119.1	C32—C31—C33	108.64 (18)
O7—C7—C4	113.41 (18)	C34—C31—Si2	110.55 (14)
O7—C7—H7D	108.9	C32—C31—Si2	112.49 (15)
C4—C7—H7D	108.9	C33—C31—Si2	108.54 (14)
O7—C7—H7C	108.9	C31—C32—H32A	109.5
C4—C7—H7C	108.9	C31—C32—H32B	109.5
H7D—C7—H7C	107.7	H32A—C32—H32B	109.5
C14—C11—C13	108.45 (17)	C31—C32—H32C	109.5
C14—C11—C12	108.59 (17)	H32A—C32—H32C	109.5
C13—C11—C12	108.09 (18)	H32B—C32—H32C	109.5
C14—C11—Si1	113.09 (15)	C31—C33—H33A	109.5
C13—C11—Si1	111.37 (14)	C31—C33—H33B	109.5
C12—C11—Si1	107.10 (14)	H33A—C33—H33B	109.5
C11—C12—H12A	109.5	C31—C33—H33C	109.5
C11—C12—H12B	109.5	H33A—C33—H33C	109.5
H12A—C12—H12B	109.5	H33B—C33—H33C	109.5
C11—C12—H12C	109.5	C31—C34—H34A	109.5
H12A—C12—H12C	109.5	C31—C34—H34B	109.5
H12B—C12—H12C	109.5	H34A—C34—H34B	109.5
C11—C13—H13A	109.5	C31—C34—H34C	109.5
C11—C13—H13B	109.5	H34A—C34—H34C	109.5
H13A—C13—H13B	109.5	H34B—C34—H34C	109.5
C11—C13—H13C	109.5	C37—C35—C36	108.51 (16)
H13A—C13—H13C	109.5	C37—C35—C38	108.29 (18)
H13B—C13—H13C	109.5	C36—C35—C38	108.12 (17)
C11—C14—H14A	109.5	C37—C35—Si2	111.45 (15)
C11—C14—H14B	109.5	C36—C35—Si2	113.31 (15)
H14A—C14—H14B	109.5	C38—C35—Si2	106.99 (13)
C11—C14—H14C	109.5	C35—C36—H36A	109.5
H14A—C14—H14C	109.5	C35—C36—H36B	109.5
H14B—C14—H14C	109.5	H36A—C36—H36B	109.5
C17—C15—C18	108.54 (17)	C35—C36—H36C	109.5
C17—C15—C16	108.67 (18)	H36A—C36—H36C	109.5
C18—C15—C16	107.65 (19)	H36B—C36—H36C	109.5
C17—C15—Si1	113.11 (16)	C35—C37—H37A	109.5
C18—C15—Si1	110.18 (14)	C35—C37—H37B	109.5
C16—C15—Si1	108.54 (15)	H37A—C37—H37B	109.5
C15—C16—H16A	109.5	C35—C37—H37C	109.5
C15—C16—H16B	109.5	H37A—C37—H37C	109.5
H16A—C16—H16B	109.5	H37B—C37—H37C	109.5
C15—C16—H16C	109.5	C35—C38—H38A	109.5
H16A—C16—H16C	109.5	C35—C38—H38B	109.5
H16B—C16—H16C	109.5	H38A—C38—H38B	109.5
C15—C17—H17A	109.5	C35—C38—H38C	109.5
C15—C17—H17B	109.5	H38A—C38—H38C	109.5
H17A—C17—H17B	109.5	H38B—C38—H38C	109.5
			
F1—Si1—C1—C2	13.58 (19)	F2—Si2—C21—C22	7.40 (19)
C15—Si1—C1—C2	126.97 (18)	C31—Si2—C21—C22	120.43 (18)
C11—Si1—C1—C2	−99.40 (18)	C35—Si2—C21—C22	−105.41 (18)
F1—Si1—C1—C6	−166.59 (17)	F2—Si2—C21—C26	−171.79 (17)
C15—Si1—C1—C6	−53.2 (2)	C31—Si2—C21—C26	−58.8 (2)
C11—Si1—C1—C6	80.4 (2)	C35—Si2—C21—C26	75.4 (2)
C6—C1—C2—C3	−0.2 (3)	C26—C21—C22—C23	−0.7 (3)
Si1—C1—C2—C3	179.61 (17)	Si2—C21—C22—C23	−179.99 (17)
C1—C2—C3—C4	0.9 (3)	C21—C22—C23—C24	0.6 (3)
C2—C3—C4—C5	−0.6 (3)	C22—C23—C24—C25	0.4 (3)
C2—C3—C4—C7	179.7 (2)	C22—C23—C24—C27	−179.0 (2)
C3—C4—C5—C6	−0.2 (3)	C23—C24—C25—C26	−1.2 (3)
C7—C4—C5—C6	179.4 (2)	C27—C24—C25—C26	178.2 (2)
C4—C5—C6—C1	0.9 (3)	C24—C25—C26—C21	1.1 (3)
C2—C1—C6—C5	−0.6 (3)	C22—C21—C26—C25	−0.1 (3)
Si1—C1—C6—C5	179.53 (17)	Si2—C21—C26—C25	179.14 (18)
C5—C4—C7—O7	14.0 (3)	C23—C24—C27—O27	−141.9 (2)
C3—C4—C7—O7	−166.4 (2)	C25—C24—C27—O27	38.8 (3)
F1—Si1—C11—C14	72.77 (15)	F2—Si2—C31—C34	−46.43 (16)
C1—Si1—C11—C14	−174.73 (14)	C21—Si2—C31—C34	−158.90 (15)
C15—Si1—C11—C14	−44.03 (17)	C35—Si2—C31—C34	70.95 (17)
F1—Si1—C11—C13	−164.80 (14)	F2—Si2—C31—C32	−167.81 (14)
C1—Si1—C11—C13	−52.30 (17)	C21—Si2—C31—C32	79.72 (17)
C15—Si1—C11—C13	78.40 (16)	C35—Si2—C31—C32	−50.43 (18)
F1—Si1—C11—C12	−46.82 (14)	F2—Si2—C31—C33	71.93 (16)
C1—Si1—C11—C12	65.68 (14)	C21—Si2—C31—C33	−40.53 (17)
C15—Si1—C11—C12	−163.61 (12)	C35—Si2—C31—C33	−170.68 (14)
F1—Si1—C15—C17	−162.48 (14)	F2—Si2—C35—C37	−166.07 (12)
C1—Si1—C15—C17	84.19 (17)	C21—Si2—C35—C37	−54.03 (15)
C11—Si1—C15—C17	−45.33 (17)	C31—Si2—C35—C37	76.69 (16)
F1—Si1—C15—C18	−40.80 (17)	F2—Si2—C35—C36	71.20 (14)
C1—Si1—C15—C18	−154.13 (14)	C21—Si2—C35—C36	−176.76 (13)
C11—Si1—C15—C18	76.35 (17)	C31—Si2—C35—C36	−46.05 (17)
F1—Si1—C15—C16	76.84 (14)	F2—Si2—C35—C38	−47.87 (15)
C1—Si1—C15—C16	−36.49 (17)	C21—Si2—C35—C38	64.17 (15)
C11—Si1—C15—C16	−166.01 (13)	C31—Si2—C35—C38	−165.11 (14)

### Hydrogen-bond geometry (Å, °)

**Table:**

*D*—H···*A*	*D*—H	H···*A*	*D*···*A*	*D*—H···*A*
O7—H7A···O27^i^	0.74 (5)	2.01 (5)	2.707 (3)	157 (5)
O27—H27A···O27^ii^	0.76 (3)	2.00 (3)	2.727 (3)	160 (5)
O7—H7B···O7^iii^	0.70 (4)	2.14 (3)	2.787 (4)	154 (5)
O27—H27B···O7^iv^	0.80 (4)	1.92 (5)	2.707 (3)	167 (4)

Symmetry codes: (i) *x*+1, *y*, *z*; (ii) −*x*, −*y*+1, −*z*+1; (iii) −*x*+1, −*y*+1, −*z*+1; (iv) *x*−1, *y*, *z*.
